# *Salmonella* Typhi-Exposed Placentae: Chorionic Villi Histomorphology and Neonatal Birthweight

**DOI:** 10.3390/diseases13070205

**Published:** 2025-06-30

**Authors:** Patience B. Tetteh-Quarcoo, Joana Twasam, Kevin Kofi Adutwum-Ofosu, John Ahenkorah, Bismarck Afedo Hottor, Nicholas T. K. D. Dayie, Peter Ofori Appiah, Emmanuel Afutu, Fleischer C. N. Kotey, Emilia Asuquo Udofia, Nii Koney-Kwaku Koney, Benjamin Arko-Boham, Eric S. Donkor

**Affiliations:** 1 Department of Medical Microbiology, University of Ghana Medical School, Accra P.O. Box KB 4236, Ghanapoappiah009@st.ug.edu.gh (P.O.A.); emmalineafutu@yahoo.com (E.A.); fcnkotey@flerholiferesearch.com (F.C.N.K.); esampane-donkor@ug.edu.gh (E.S.D.); 2 Department of Anatomy, University of Ghana Medical School, Accra P.O. Box KB 4236, Ghana; kadutwum-ofosu@ug.edu.gh (K.K.A.-O.); nkkoney@ug.edu.gh (N.K.-K.K.); barko-boham@ug.edu.gh (B.A.-B.); 3 FleRhoLife Research Consult, Teshie, Accra P.O. Box TS 853, Ghana; 4 Department of Community Health, University of Ghana Medical School, Accra P.O. Box KB 4236, Ghana; eudofia@ug.edu.gh

**Keywords:** *Salmonella* Typhi, placental histomorphology, birthweight, stereology, pregnancy complications

## Abstract

Background: *Salmonella* infections impose a substantial global health burden, with an estimated 95.1 million cases occurring annually. Pregnant women exhibit a heightened vulnerability due to pregnancy-specific immune adaptations and dietary habits that increase their risk of *Salmonella* exposure, facilitating possible damage to the placental barrier. Despite this significant burden, *Salmonella*-associated placental pathology remains poorly understood, particularly its impact on foetal development through microstructural alterations. Aim: This study utilised stereology to assess histomorphological and functional alterations in term placentae of *Salmonella* Typhi-exposed placentae, compared to unexposed controls. Methods: A hospital-based case-control study was conducted in Ghana. Of 237 screened women, 62 placentae were selected for analysis, comprising 31 *Salmonella*-exposed cases (IgG/IgM-positive in placental and cord blood) and 31 gestational age-matched controls (IgG/IgM-negative). Placental tissues were processed for histology and stereology. Neonatal birthweights were also compared. Results: Stereological assessment revealed significantly higher mean volume densities of syncytial knots in the study group (0.4755 ± 0.04) compared to the controls (0.3342 ± 0.04, *p* = 0.0219). Syncytial denudation was increased in the study group (0.8113 ± 0.09) relative to the controls (0.1975 ± 0.08, *p* < 0.0001). Foetal capillary volume density was also significantly elevated in the study group (5.1010 ± 0.32) compared to the controls (3.562 ± 0.47, *p* < 0.0001). In contrast, intervillous space volume was significantly reduced in the study group (9.5810 ± 0.05) compared to the controls (11.593 ± 0.26, *p* = 0.0053). Neonates of exposed mothers showed a non-significant reduction in birthweight. Conclusion: *Salmonella* Typhi exposure in pregnancy induces subtle, yet significant alterations in placental architecture, compromising villous integrity and vascular organisation. Although birthweight may appear unaffected, the observed changes point to reduced placental efficiency and merit further research into their developmental consequences and long-term effects on babies.

## 1. Introduction

*Salmonella* infection is one of the most important foodborne illnesses worldwide, including in Ghana [1]. The Global Burden of Diseases, Injuries, and Risk Factors Study (GBD), estimated the cases of *Salmonella* infection to have numbered 95.1 million, resulting in 50,771 deaths in 2017 [2]. The illness is normally self-limiting and non-severe, with symptoms resolving within a mean of 4–7 days [3,4]. Complications such as reactive arthritis or secondary infection in other organs can occur, mainly in young children, the elderly, pregnant women, or immunocompromised persons [5].

Immunological adaptations occur in pregnancy, including the shift toward a Th2-dominant response and a shift away from Thl responses. This bias reduces Th1 cytokines, impairing the recruitment and activation of innate immune cells (e.g., NK cells and neutrophils) necessary to control bacterial infections, while elevated IL-6 levels further disrupt protective immune coordination, thereby increasing maternal susceptibility to typhoid fever [6,7,8]. In animal models, *Salmonella* doses as low as 10^2^ CFU administered during late gestation have been found to alter the placental morphometry, inducing placenta inflammation that leads to negative consequences in pregnancy outcomes, such as significant reduction in foetal body weight [3,9]. *Salmonella* infection can also lead to pregnancy complications such as chorioamnionitis, transplacental foetal infection, spontaneous abortion, and both neonatal and maternal septicemia [10,11]. However, the specific mechanisms by which *Salmonella* infections trigger placental pathology is not clear.

The placenta is a composite organ made of foetal and maternal tissues, serving as both a physical and biological barrier that protects the foetus from pathogens [12]. The placenta may be regarded as a metabolic mirror of a mother and foetus’s health status [13]. The organ responds to the environment in an attempt to ensure foetal viability. Hence, detailed macroscopic and microscopic descriptions of the placenta are important in grasping the process of gestation pathophysiology. Any disruption of placental development, growth, or function by disease can significantly compromise the well-being of both the mother and foetus [14]. Placental structure plays a central role in sustaining foetal development, and even subtle morphological changes can have important physiological consequences. *Salmonella* Typhi infection may alter placental microarchitecture in ways that are not immediately readily detectable through clinical outcomes alone. This study, therefore, examined specific histological features—syncytial knots, syncytial denuded areas, foetal capillaries, and intervillous gaps—that are closely linked to placental efficiency in oxygen and nutrient exchange [12]. We aimed to quantitatively assess these features in term placentae from *S.* Typhi-exposed and unexposed pregnancies using stereological methods. To the best of our knowledge, this is the first study to explore how maternal exposure to *Salmonella* Typhi affects the microscopic architecture of the human term placenta. It offers new insights into subtle structural vulnerabilities that may occur even in the absence of overt clinical symptoms.

## 2. Materials and Methods

### 2.1. Study Design, Location, and Sample Collection

This case-control study involved the purposive sampling of postpartum placentae from women who delivered at two healthcare facilities in the Greater Accra Region of Ghana: LEKMA Hospital and Weija-Gbawe Municipal Hospital, both of which are municipal hospitals in Accra. Participants were recruited antenatally, and written informed consent was obtained before delivery. Placental samples were obtained from the Obstetrics and Gynaecology Departments of the selected hospitals. This study included only term pregnancies, defined as those reaching 38 ± 2 weeks of gestation. Eligible placentae were obtained from women who delivered either by spontaneous vaginal delivery or by cesarean section. Gestational age was estimated using the last menstrual period as recorded in maternal health records, and the neonatal birthweights were documented. Immediately following delivery, each placenta was placed in a sterile kidney dish and then transferred to a larger sterile container for examination. Placental assessment followed the procedure outlined by Kaplan [15], which involves examining the placenta for shape, completeness of membranes, cord insertion, and gross lesions such as infarcts, hematomas, or calcifications.

### 2.2. Study Groups and Classification

Blood samples were obtained from two sites: basal placental (maternal side) and umbilical cord (foetal side). A volume of 2–3 mL of blood was drawn from each site into K2EDTA tubes (BD Vacutainer^®^, Becton, Dickinson & Company (BD), Plymouth, UK) for *Salmonella* Typhi rapid diagnostic testing. Participants were categorised into either the case (study) group or the control group based on the presence or absence of *Salmonella* Typhi antibodies (IgG and IgM) in samples obtained from the placental basal plate and umbilical cord. Samples that tested positive for both IgG and IgM antibodies at both anatomical sites were classified as cases, while those that tested negative (IgG and IgM) for both antibodies at both sites were designated as controls. To minimise the influence of potential confounders, we carefully reviewed patients’ clinical records. Particular attention was given to comorbidities and exposure to other endemic pathogens in the region, such as HIV, Hepatitis B and C, *Plasmodium* spp., *Toxoplasma gondii*, and *Treponema pallidum*. We also accounted for genetic disorders such as sickle cell disease, as well as various maternal clinical factors verified through medical documentation. Only placentae from women who tested positive for *Salmonella* Typhi antibodies and negative for all other screened pathogens were included in the case group. Controls consisted of placentae that tested negative for exposure to all screened pathogens, including *Salmonella* Typhi.

### 2.3. Serological Testing for Salmonella Typhi

Exposure to *Salmonella* Typhi was assessed using the JusChek Typhoid IgG/IgM Rapid Test Cassette (ACRO Biotech, Inc., Montclair, CA, USA, Catalogue No. ITY-402), a qualitative immunoassay designed to detect *S.* Typhi IgG and IgM antibodies in blood, serum, or plasma. Each test cassette was removed from its packaging and placed on a clean, flat surface, then labeled with the corresponding specimen identification number. Approximately 40 µL of blood was added to the sample well, followed by a drop of buffer reagent. The immunocomplex formed between any present antibodies and the precoated *Salmonella* Typhi antigens migrated through the membrane by capillary action. Results were interpreted after 15–20 min. A negative (non-reactive) result was defined by the presence of only the control (C) band, with no visible burgundy colouration in the IgM (M) or IgG (G) test lines—indicating the absence of *S.* Tphi antibodies. A positive (reactive) result was indicated by the appearance of the C band along with a burgundy line at the M band (indicating IgM antibodies), the G band (indicating the presence of IgG antibodies), or both (indicating the presence of both antibody classes).

### 2.4. Placental Tissue Collection and Processing

Each placenta was thoroughly rinsed under running tap water to remove residual blood before to gross morphological assessment. To ensure systematic and representative tissue sampling, each placenta was divided into four quadrants. From these, four full-thickness tissue sections (measuring approximately 2 cm × 2 cm × 5 cm) were obtained, spanning from the chorionic plate to the basal plate. Sampling locations were strategically selected to capture spatial variation: the first sample was taken near the umbilical cord insertion, the second at an intermediate distance from the cord, the third near the periphery, and the fourth at the placental edge. This protocol ensured balanced representation of villous architecture across central and peripheral zones of the placental disc.

Immediately after collection, tissue samples were fixed in 10% phosphate-buffered formalin (pH 7.24–7.28) to preserve morphology and prevent autolysis. Fixed tissues were then processed using an automated tissue processor (LEICA TP 1020, Wetzlar, Germany). The processing involved dehydration through a graded ethanol series (70%, 80%, 95%, and 100%), followed by two sequential xylene treatments (1 h each) for clearing. Tissue infiltration was completed using molten paraffin wax at 58 °C. Finally, the samples were embedded in paraffin blocks and allowed to cool, rendering them suitable for microtomy and subsequent histological analysis.

### 2.5. Tissue Sectioning and Staining

Microtomy was conducted using a Leica RM 2125RT rotary microtome (Leica Biosystems, Wetzlar, Germany). Paraffin-embedded placental tissues were first trimmed to a thickness of 10 µm to expose the full tissue profile. Subsequently, thin sections of 5 µm were obtained and mounted on standard-sized glass microscope slides (76 mm × 26 mm × 1 mm). To ensure consistent spatial representation, four sections per tissue block—specifically the 1st, 50th, 100th, and 150th sections—were selected for hematoxylin and eosin (H&E) staining (Merck KGaA, Darmstadt, Germany). The staining protocol involved deparaffinization in xylene, followed by rehydration through descending ethanol concentrations. Sections were stained with hematoxylin to visualize nuclei, counterstained with eosin to delineate cytoplasmic and extracellular components, then dehydrated and mounted using Distyrene Plasticizer Xylene (DPX) (Merck KGaA) to preserve the samples for light microscopy.

### 2.6. Stereological Investigations

#### 2.6.1. Sampling of Photomicrographs of Placental Sections

A total of 62 placental tissue sections (31 cases and 31 controls) were examined under an optical light microscope (Leica Galen III, Catalogue No. 317506, Serial No. ZG6JA4, Wetzlar, Germany) equipped with a 40× objective lens. For digital imaging, the microscope’s standard eyepiece was replaced with a Lenovo Q350 USB digital camera (Beijing, China), which was connected to an HP Compaq dx2300 Microtower desktop system (HP Inc., Palo Alto, CA, USA) to enable digital image acquisition. To ensure systematic imaging, the field of view was initially positioned at one corner of the basal plate. The microscope stage was then shifted in a controlled, grid-like manner—two units along the x-axis and two units along the y-axis—before capturing each image. For subsequent rows, the stage was moved in the reverse direction along the x-axis while maintaining the same direction along the y-axis, forming a zigzag scanning pattern that allowed comprehensive coverage of the entire slide. All photomicrographs were captured at 40× magnification using the digital camera, yielding an average of 50 images per placental slide.

**Figure 1:**
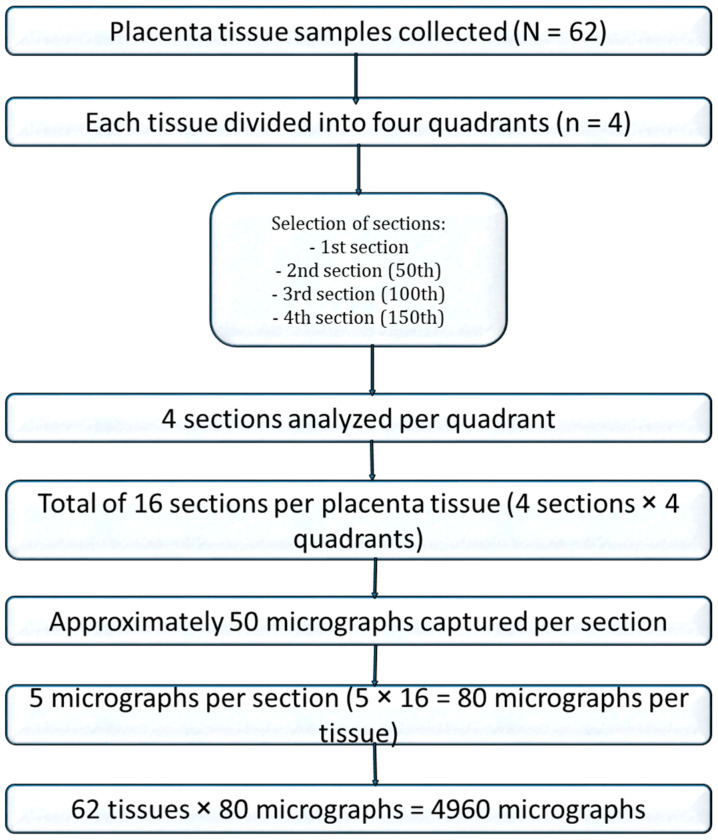
Flow chart of placenta tissue selection for stereology.

For stereological analysis, images from 16 sections per placenta were compiled. A systematic random sampling approach was used: after a random starting point, every third photomicrograph was selected from the sequence, generating 80 images per placenta and a total of 4960 photomicrographs for quantitative analysis (Figure 1).

To ensure accurate calibration, images of a microscope-stage graticule captured at the same magnification were used to adjust the stereological grid. Hematoxylin and eosin (H&E)-stained sections were analyzed using stereological point-counting methods to obtain quantitative data on key histological features, including syncytial knots, syncytial necrosis, intervillous spaces, and foetal capillaries.

#### 2.6.2. Stereological Analysis of Placental Photomicrographs

A design-based stereological approach was employed to determine the volume densities of key structural parameters, including syncytial knots, syncytial denudation, intervillous spaces, and foetal capillaries, using point counting with the Cavalieri principle [16], which estimates volume by summing measurements from systematically spaced tissue sections. Adobe Photoshop CS6 Extended (trial version 13.0.1) software was used to overlay a calibrated 1 cm × 1 cm stereological grid onto each photomicrograph. Grid points falling on the features of interest were counted, and relative volume densities were computed using Cavalieri’s principle with the following formula:

Vv = ∑P × (ap) × t/M2

where Vv represents volume density, ∑P is the total number of grid points counted, ap is the area per point, t is the section thickness, and M is the linear magnification [17].

### 2.7. Statistical Analysis

Statistical analysis was performed using GraphPad Prism (version 5, GraphPad Software LLC of Boston, MA, USA) after data were entered into Microsoft Excel 2019 (version 16, Microsoft Corporation, Redmond, WA, USA). Descriptive statistics, including means, standard deviations (SDs), standard errors of the mean (SEM), and 95% confidence intervals (CIs), were calculated for all quantitative variables. Group comparisons of mean volume densities were conducted using unpaired *t*-tests and one-way analysis of variance (ANOVA). Bartlett’s test was applied to assess the assumption of equal variances across groups. A *p*-value of ≤0.05 was considered statistically significant.

### 2.8. Ethical Consideration

Ethical approval for the study was obtained (approval date: 1 June 2020) from the Ethical and Protocol Review Committee of the College of Health Sciences, University of Ghana (CHS-Et/M4-P.7/2019-2020), with permissions also granted by Weija-Gbawe Municipal Hospital and LEKMA Hospital. All procedures involving human participants were reviewed and approved by the appropriate institutional and national ethics committees, and conducted in accordance with the ethical standards outlined in the Declaration of Helsinki, as revised in 2013 [18].

## 3. Results

### 3.1. Sample Characteristics

Out of the 237 placental specimens collected from the two municipal hospitals, a total of 474 blood samples were obtained from the umbilical cord and placental basal plate for serological analysis. The detection of *Salmonella* Typhi IgG and IgM antibodies in both cord and placental basal plate blood was observed in 62/474 blood samples taken from 31/237 (13.08%) placental tissue specimens, thereby fulfilling the case definition for *Salmonella* Typhi-exposed placentae (cases) matched with the selected 31 IgG- and IgM-negative controls, leading to the 62 placental tissues selected for the histomorphological and stereological part of the study.

### 3.2. Placental Histomorphological Parameters

**Figure 2:**
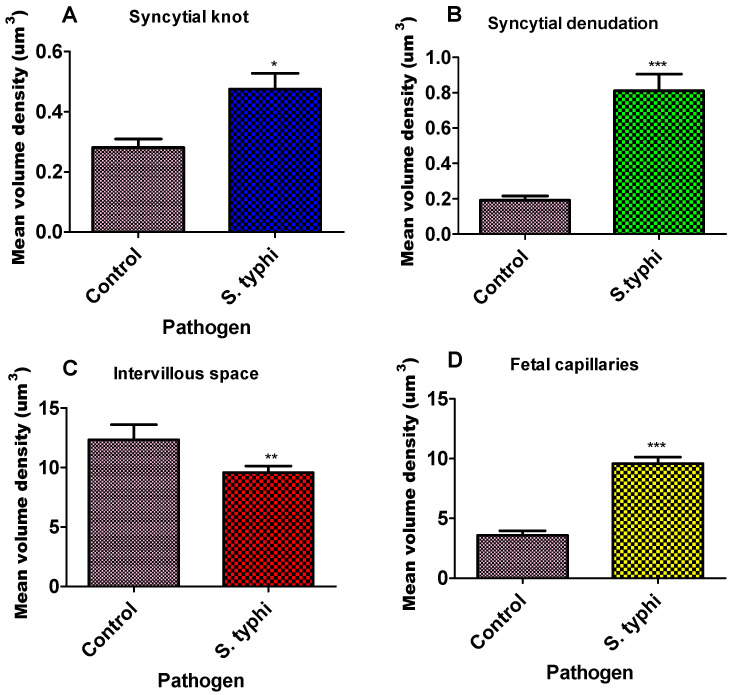
Bar graph of the mean volume density of placenta parameters for *Salmonella* Typhi. Each column represents the mean ± S.E.M. Unpaired T-test with * *p* ≤ 0.05, ** *p* ≤ 0.01, and *** *p* ≤ 0.001 compared to their matched pairs. (**A**)—syncytial knot, (**B**)—syncytial denudation, (**C**)—intervillous space, and (**D**)—foetal capillaries for *Salmonella* Typhi.

Unpaired *t*-test analyses of the placentae at term showed significant differences between the study group and the control group (Table 1, Figure 2).

The volume densities of syncytial knots, foetal capillaries, and syncytial denuded areas were significantly higher in the study group compared to the controls (Figure 2, Table 1). Specifically, the mean volume density of syncytial knots was markedly elevated in the study group (0.4755 ± 0.04) relative to the controls (0.3342 ± 0.04; *p* = 0.0219). Syncytial denudation was also significantly greater in the study placentae (0.8113 ± 0.09) than in the control group (0.1975 ± 0.08; **** *p* < 0.0001). Similarly, foetal capillary volume density was increased in the study group (5.1010 ± 0.32) compared to the controls (3.562 ± 0.47; **** *p* < 0.0001). In contrast, the intervillous space was significantly reduced in the exposed placentae (9.5810 ± 0.05) relative to the control group (11.593 ± 0.26; *p* = 0.0053).

**Figure 3:**
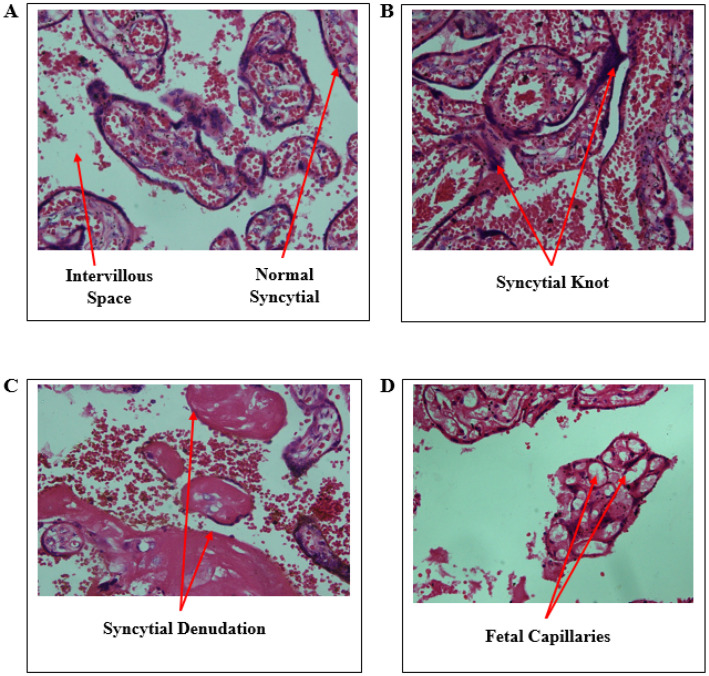
Micrographs of *Salmonella* Typhi-exposed and unexposed placentae. (**A**)—control with normal syncytial, (**B**)—syncytial knot, (**C**)—syncytial denudation, and (**D**)—foetal capillaries and intervillous space.

Micrographs of placenta sections stained with hematoxylin and eosin are shown in Figure 3. The histology of the control shows a normal syncytium of the placenta with single cells lining the syncytium. However, some small amount of the knot is seen in the control, but the morphology of the cell is maintained on the syncytium. *Salmonella* Typhi had a significant syncytial denudation.

**Figure 4:**
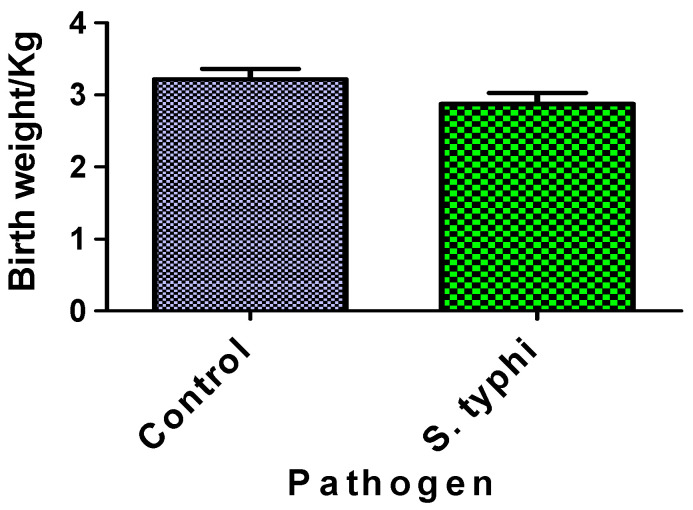
Birthweight of babies from case and control mothers. Each column represents the mean ± S.E.M.

Figure 4 compares neonatal birthweights between *Salmonella* Typhi-exposed cases and unexposed controls. The difference in birthweights between the two groups was not statistically significant. Both groups’ birthweight distributions fell within normal clinical ranges.

## 4. Discussion

Our findings demonstrate a significant increase in syncytial knot density in *Salmonella* Typhi-exposed placentae compared to *Salmonella* Typhi-non-exposed placentae. These pathological alterations likely reflect trophoblast stress induced by hypoxia, potentially mediated by infection-related vascular dysfunction and endothelial injury. Hypoxia is a major driver of excessive syncytial knotting, often resulting from inadequate maternal blood flow to the placenta. The observed changes are reminiscent of placental adaptations seen in other conditions of impaired uteroplacental perfusion, including preeclampsia, intrauterine growth restriction, certain infections, as well as diseases such as malaria and sickle cell disease [14,19,20,21]. The parallel is striking because *Salmonella* infection is known to trigger two key harmful processes: first, it causes widespread inflammation that can injure the delicate placental blood vessels, and second, it releases damaging molecules called cytokines that further disrupt normal function [22]. While our study design cannot establish a direct causal link between these histological changes and adverse foetal outcomes, the presence of these characteristic stress markers suggests that *Salmonella* infection may compromise placental function through pathways similar to other clinically significant pregnancy complications. Clinically, increased syncytial knots are associated with pregnancy complications such as preeclampsia and foetal growth restriction, conditions marked by poor placental circulation and reduced foetal nutrient supply [23,24]. Detached syncytial knots may also accumulate in the intervillous space, contributing to systemic maternal inflammation and worsening pregnancy outcomes [25,26].

Another change that occurred in the syncytium was the presence of denuded regions (nuclei completely removed or abraded) areas in the placenta, where there was a significant increase in placentae infected with *Salmonella* Typhi. The syncytiotrophoblast is the outermost layer of the placental villi, responsible for nutrient and gas exchange between the mother and foetus. This observation reflects extensive damage to the syncytiotrophoblast, the placenta’s critical barrier and functional interface. Widespread denudation exposes the underlying villous stroma, compromises nutrient transport, and increases foetal vulnerability to maternal pathogens and inflammatory mediators. The mechanisms likely involve *Salmonella*’s unique pathogenic strategies. Gram-negative bacteria like *Salmonella* trigger apoptosis in trophoblast cells through Toll-like receptor 4 (TLR4)-mediated inflammation [27] and lipopolysaccharide (LPS)-induced oxidative stress [28]. In response to *Salmonella* infection, elevated levels of pro- and anti-inflammatory cytokines, including IFN-γ, TNF-α, IL-17, and IL-10 have been detected in the placenta, amniotic fluid, and maternal serum [29]. Increased placental IFN-γ expression has been associated with placental damage and the upregulation of infiltration and hypoxia markers, including cyclooxygenase-1 (COX-1) and cyclooxygenase-2 (COX-2), which may contribute to adverse pregnancy outcomes such as preterm birth [3,30]. Additionally, heightened IL-17 levels may be detrimental to pregnancy maintenance and may promote inflammation at the foetal–maternal interface [3,31]. Our findings align with these molecular studies, providing histological evidence of *Salmonella*’s placental tropism in clinical cases. The exposed placentae had a significant increased volume density of villous syncytial denuded areas, and this may suggest changes in villous maturation that may have been caused by the exposure of the *Salmonella* to the placenta. This observation of the current study implies that the structural integrity of the placental blood barrier has been greatly compromised with the presence or exposure to *Salmonella*. Maternal immune cells such as immunoglobulin G (IgG) can easily cross the barrier [32] and give immunity to the foetus. However, in some instances where immunoglobulin M (IgM) is able to cross the blood barrier, then it means that the defense of the blood barrier has been removed to allow for higher-molecular-weight immunoglobulin, such as the IgM, to be found in the cord blood. Because the foetal–maternal barrier has been breached by syncytial denudation, blood from the intervillous space may easily reach the foetal capillaries for bacteria present in the maternal blood to be easily transmitted to the foetus.

Intervillous spaces of the placenta are where the maternal blood is found, and are also the maternal spaces through which oxygen molecules can reach the foetus. Thus, it is very important to mention that there was a significant decrease in placentae infected with *S.* Typhi. Ernst [33] reported that IUGR correlates with significantly lower intervillous space. This reduction may also reflect villous congestion or excessive trophoblastic proliferation, both of which can compromise maternal blood flow, trigger placental hypoxia, and increase the risk of foetal demise or preterm birth [34]. The results of the intervillous space can be compared with other research where areas of intervillous space appeared to be smaller or significantly reduced in the *Plasmodium*-exposed groups when looking at pregnant women exposed to *Plasmodium vivax* [35,36]. Also, there has been report of a significant decrease in villous areas in active malaria-infected cases compared to both controls and treated malaria cases [37]. Increasing the sizes of intervillous pores is expected to facilitate the flow of maternal blood [38], but the decrease in this instance means the amount of blood flowing from the mother is reduced, leading to placental hypoxia, which has been complemented by an observed increase in foetal capillaries in this study.

Despite the marked stereological and histomorphological disruptions in *Salmonella*-exposed placentae, no significant differences in neonatal birthweights were observed between the exposed and control groups. This paradox likely reflects the human placenta’s known functional reserve capacity; some percentage of villous tissue can be compromised before clinical growth restriction emerges [39,40]. The completion of full-term gestation in our term-delivery cohort may have also permitted compensatory foetal adaptations, contrasting with the more-severe impacts typically seen with early-gestation deliveries [41,42]. This discordance between placental pathology and foetal growth weight also shows the multifactorial regulation of birthweight, which integrates maternal nutrition, genetic determinants, metabolic adaptations, and compensatory placental mechanisms. It is observed in a study that nearly 8% of the infants born during the study period had birthweights below 2.5 kg (LBW) and was associated with poor socioeconomic status and maternal malnutrition [43]. Because this study did not assess socioeconomic status and maternal malnutrition, the low birthweight recorded in this study cannot be attributed to that effect.

The absence of growth restriction despite structural anomalies may reflect functional redundancy in placental nutrient transport (e.g., the upregulation of glucose transporters or hemodynamic adjustments) or temporal resilience, wherein late-term infections permit the recovery of foetal growth trajectories. Alternatively, the observed architectural changes, while statistically significant, may not have exceeded the threshold required to disrupt gross foetal development. Critically, these findings align with emerging evidence that subclinical infections can induce subcellular or molecular placental alterations without overt clinical phenotypes [44,45,46]. These results emphasise that birthweight, as a composite endpoint, may lack sensitivity to specific placental insults unless compounded by additional stressors (e.g., maternal malnutrition or concurrent infections), highlighting the need for multidimensional assessment in future research. Further studies integrating Doppler flow metrics, placental transcriptomics, and longitudinal growth monitoring are warranted to dissect these compensatory pathways.

### Limitation

The study did not include a follow-up of neonates born to infected mothers. This limits our ability to determine how these placental alterations translate into long-term neonatal health outcomes, including growth, immune function, and susceptibility to infections. Future research incorporating maternal clinical profiles, longitudinal neonatal follow-up, and functional assessments of placental efficiency would be essential to fully understand the clinical implications of *Salmonella* Typhi exposure during pregnancy.

## 5. Conclusions

*Salmonella* Typhi exposure in pregnancy leads to distinct placental alterations marked by increased syncytial knotting, enhanced foetal capillarisation, and reduced intervillous space, indicating structural stress and adaptive remodeling. These changes occurred despite normal birthweights, suggesting that standard perinatal metrics might not truly reflect the state of the placenta. This highlights the importance of improving maternal infection surveillance in endemic regions. Understanding these placental changes may provide a basis for earlier risk identification and improved neonatal outcomes. There is the need to further investigate the developmental and clinical implications of infection-induced placental injury, particularly in regions where *Salmonella* remains endemic.

## Figures and Tables

**Table 1:** Volume density (Vd) of placental parameters among the study and control placenta groups.

*Salmonella* Typhi				
Variable	Control	Study	*t*-Value	*p*-Value
Syncytial knot	0.3342 ± 0.04	0.4755 ± 0.04	1.820	0.0219
Syncytial denudation	0.1975 ± 0.08	0.8113 ± 0.09	5.066	<0.0001
Intervillous spaces	11.593 ± 0.26	9.5810 ± 0.05	2.278	0.0053
Foetal capillaries	3.562 ± 0.47	5.1010 ± 0.32	3.008	0.0001

The presented values indicate the mean ± SEM (standard error of the mean), with accompanying *p*-values indicating the significance level for one-way analysis of variance (followed by Tukey’s post hoc analysis).

## Data Availability

The data presented in this study are available upon reasonable request from the corresponding authors (patborket2002@yahoo.com or pbtetteh-quarcoo@ug.edu.gh and jahenkorah@ug.edu.gh).

## References

[1] Andoh L.A., Ahmed S., Olsen J.E., Obiri-Danso K., Newman M.J., Opintan J.A., Barco L., Dalsgaard A. (2017). Prevalence and characterization of *Salmonella* among humans in Ghana. Trop. Med. Health.

[2] Bassat Orellana Q. (2019). The global burden of non-typhoidal *Salmonella* invasive disease: A systematic analysis for the Global Burden of Disease Study 2017. Lancet Infect. Dis..

[3] Betancourt D.M., Noto Llana M., Sarnacki S.H., Cerquetti M.C., Monzalve L.S., Pustovrh M.C., Giacomodonato M.N. (2021). *Salmonella* Enteritidis foodborne infection induces altered placental morphometrics in the murine model. Placenta.

[4] Lenchenko E.M., Vatnikov Y.A., Kulikov E.V., Lozovoi D.A., Gavrilov V.A., Gnezdilova L.A., Zimina V.N., Kuznetsov V.I., Annikov V.V., Medvedev I.N. (2019). Aspects of Salmonellosis pathogenesis using chicken models. Bali Med. J..

[5] Trejo-Ruiz L.T., Guzmán Martínez N., Ruvalcaba Ledezma J.C. (2015). Knowledge of mechanisms of transmission and complications of salmonellosis in over 18 years of Pachuca, Hidalgo, Mexico. Biomed. Pharmacol. J..

[6] Chattopadhyay A. (2009). Role of *Salmonella* Typhimurium Virulence in Differentially Modulating Immune Response and Host Susceptibility During Pregnancy. Ph.D. Thesis.

[7] Coe C.L., Lubach G.R. (2007). Mother-infant interactions and the development of immunity from conception through weaning. Psychoneuroimmunology.

[8] Shukla G., Verma I., Sharma L. (2012). Effect of *Salmonella enteric* serovar Typhimurium in pregnant mice: A biochemical and histopathological study. Gastroenterol. Res..

[9] Chattopadhyay A., Robinson N., Sandhu J.K., Finlay B.B., Sad S., Krishnan L. (2010). *Salmonella enterica* Serovar Typhimurium-Induced Placental Inflammation and Not Bacterial Burden Correlates with Pathology and Fatal Maternal Disease. Infect. Immun..

[10] Hedriana H.L., Mitchell J.L., Williams S.B. (1995). *Salmonella* typhi chorioamnionitis in a human immunodeficiency virus-infected pregnant woman. A case report. J. Reprod. Med..

[11] Schloesser R.L., Schaefer V., Groll A.H. (2004). Fatal Transplacental Infection with Non-typhoidal *Salmonella*. Scand. J. Infect. Dis..

[12] Heerema-McKenney A. (2018). Defense and infection of the human placenta. APMIS.

[13] Resta L., Rossi R., Fulcheri E., Malvasi A., Tinelli A., Di Renzo G.C. (2017). The Placenta as the Mirror of the Foetus. Management and Therapy of Late Pregnancy Complications.

[14] Sankar K.D., Bhanu P.S., Ramalingam K., Kiran S., Ramakrishna B.A. (2013). Histomorphological and morphometrical changes of placental terminal villi of normotensive and preeclamptic mothers. Anat. Cell Biol..

[15] Kaplan C. (2013). Gross examination of the placenta. Surg. Pathol. Clin..

[16] Dezfoolian A., Panahi M., Feizi F. (2009). Stereological evaluation of renal glomeruli in offspring of diabetic female rats. Cell J..

[17] Heidari Z., Sakhavar N., Mahmoudzadeh-Sagheb H., Ezazi-Bojnourdi T. (2015). Stereological analysis of human placenta in cases of placenta previa in comparison with normally implanted controls. J. Reprod. Infertil..

[18] World Medical Association (2013). World Medical Association Declaration of Helsinki: Ethical principles for medical research involving human subjects. JAMA.

[19] Ahenkorah J., Tetteh-Quarcoo P.B., Nuamah M.A., Kwansa–Bentum B., Nuamah H.G., Hottor B., Korankye E., Torto M., Ntumy M., Addai F.K. (2019). The Impact of *Plasmodium* Infection on Placental Histomorphology: A Stereological Preliminary Study. Infect. Dis. Obstet. Gynecol..

[20] Burton G.J., Jauniaux E. (2018). Pathophysiology of placental-derived fetal growth restriction. Am. J. Obstet. Gynecol..

[21] Mumuni M., Adutwum-Ofosu K.K., Arko-Boham B., Hottor B.A., Koney N.K.-K., Adu-Bonsaffoh K., Oppong S.A., Appiah P.O., Ahenkorah J. (2025). Histomorphology of placentae of women with sickle cell disease during pregnancy–A case control study. PLoS ONE.

[22] Dougan G., Baker S. (2014). *Salmonella enterica* Serovar Typhi and the Pathogenesis of Typhoid Fever. Annu. Rev. Microbiol..

[23] Mousa B.A., Al Joborae S.F. (2019). Study of placental shape and histopathological changes in pregnant ladies with preeclampsia. Iraq Med. J..

[24] Saeed I., Iqbal I., Sarfaraz R., Qamar K., Butt S.A., Shaukat S. (2012). Histomorphological changes in placentae of preeclamptic mothers with reference to vasculosyncytial membrane thickness and syncytial knot formation. J. Rawalpindi Med. Coll..

[25] Cohen M.C., Scheimberg I., Hunson J.C., Baergen R.N., Burton G.J., Kaplan C.G. (2022). Anatomy and Pathology of the Placental Membranes. Benirschke’s Pathology of the Human Placenta.

[26] Roland C.S., Hu J., Ren C.-E., Chen H., Li J., Varvoutis M.S., Leaphart L.W., Byck D.B., Zhu X., Jiang S.-W. (2016). Morphological changes of placental syncytium and their implications for the pathogenesis of preeclampsia. Cell. Mol. Life Sci..

[27] Nguyen T. (2017). Mode of Entry and Survival of *Salmonella enterica* Serovar Typhimurium in Trophoblast Cells. Ph.D. Thesis.

[28] Zhu Q., Han Y., Wang X., Jia R., Zhang J., Liu M., Zhang W. (2023). Hypoxia exacerbates intestinal injury and inflammatory response mediated by myeloperoxidase during *Salmonella* Typhimurium infection in mice. Gut Pathog.

[29] Noto Llana M., Sarnacki S.H., Aya Castañeda M.d.R., Pustovrh M.C., Gartner A.S., Buzzola F.R., Cerquetti M.C., Giacomodonato M.N. (2014). *Salmonella enterica* serovar Enteritidis enterocolitis during late stages of gestation induces an adverse pregnancy outcome in the murine model. PLoS ONE.

[30] Reese J., Paria B.C., Brown N., Zhao X., Morrow J.D., Dey S.K. (2000). Coordinated regulation of fetal and maternal prostaglandins directs successful birth and postnatal adaptation in the mouse. Proc. Natl. Acad. Sci. USA.

[31] Liu B., Zhang X., Ding X., Bin P., Zhu G. (2023). The vertical transmission of *Salmonella* Enteritidis in a one-health context. One Health.

[32] Palmeira P., Quinello C., Silveira-Lessa A.L., Zago C.A., Carneiro-Sampaio M. (2012). IgG placental transfer in healthy and pathological pregnancies. Clin. Dev. Immunol..

[33] Ernst L.M. (2018). Maternal vascular malperfusion of the placental bed. APMIS.

[34] Ravishankar S., Redline R.W. (2019). The placenta. Handb. Clin. Neurol..

[35] Souza R.M., Ataíde R., Dombrowski J.G., Ippólito V., Aitken E.H., Valle S.N., Álvarez J.M., Epiphânio S., Marinho C.R.F. (2013). Placental Histopathological Changes Associated with Plasmodium vivax Infection during Pregnancy. PLoS Neglected Trop. Dis..

[36] Brabin B.J., Romagosa C., Abdelgalil S., Menéndez C., Verhoeff F.H., McGready R., Fletcher K.A., Owens S., d’Alessandro U., Nosten F. (2004). The sick placenta—The role of malaria. Placenta.

[37] Chaikitgosiyakul S., Rijken M.J., Muehlenbachs A., Lee S.J., Chaisri U., Viriyavejakul P., Turner G.D., Pongponratn E., Nosten F., McGready R. (2014). A morphometric and histological study of placental malaria shows significant changes to villous architecture in both Plasmodium falciparum and Plasmodium vivax infection. Malar. J..

[38] Rainey A., Mayhew T.M. (2010). Volumes and Numbers of Intervillous Pores and Villous Domains in Placentas Associated with Intrauterine Growth Restriction and/or Pre-eclampsia. Placenta.

[39] Kingdom J., Huppertz B., Seaward G., Kaufmann P. (2000). Development of the placental villous tree and its consequences for fetal growth. Eur. J. Obstet. Gynecol. Reprod. Biol..

[40] Zhang S., Regnault T.R., Barker P.L., Botting K.J., McMillen I.C., McMillan C.M., Roberts C.T., Morrison J.L. (2015). Placental adaptations in growth restriction. Nutrients.

[41] Zhao Y., Zhang W., Tian X. (2022). Analysis of risk factors of early intraventricular hemorrhage in very-low-birth-weight premature infants: A single center retrospective study. BMC Pregnancy Childbirth.

[42] Diabelková J., Rimárová K., Urdzík P., Dorko E., Houžvičková A., Andraščíková Š., Drabiščák E., Škrečková G. (2022). Risk factors associated with low birth weight. Cent. Eur. J. Public Health.

[43] GSS, GHS, ICF (2014). International Ghana demographic health survey. Demogr. Health Surv..

[44] Wang J., Qian R., Wang Y., Dong M., Liu X., Zhou H., Ye Y., Chen G., Chen D., Yuan L. (2021). The mediation effect of placental weight change in the association between prenatal exposure to selenium and birth weight: Evidence from a prospective birth cohort study in China. Environ. Epidemiol..

[45] Agarwal N., Papanna R., Sibai B.M., Garcia A., Lai D., Soto Torres E.E., Amro F.H., Blackwell S.C., Hernandez-Andrade E. (2025). Evaluation of fetal growth and birth weight in pregnancies with placenta previa with and without placenta accreta spectrum. J. Perinat. Med..

[46] Hietalati S., Pham D., Arora H., Mochizuki M., Santiago G., Vaught J., Lin E.T., Mestan K.K., Parast M., Jacobs M.B. (2024). Placental pathology and fetal growth outcomes in pregnancies complicated by maternal obesity. Int. J. Obes..

